# Organic molecules with inverted singlet-triplet gaps

**DOI:** 10.3389/fchem.2022.999856

**Published:** 2022-08-24

**Authors:** Jie Li, Zhi Li, Hui Liu, Heqi Gong, Jincheng Zhang, Yali Yao, Qiang Guo

**Affiliations:** ^1^ College of Optoelectronic Engineering, Chengdu University of Information Technology, Chengdu, China; ^2^ School of Physics and Engineering Technology, Chengdu Normal University, Chengdu, China

**Keywords:** inverted singlet-triplet, down conversion, organic light-emitting materials, reverse intersystem crossing, thermal activated delayed fluorescence

## Abstract

According to Hund’s multiplicity rule, the energy of the lowest excited triplet state (T_1_) is always lower than that of the lowest excited singlet state (S_1_) in organic molecules, resulting in a positive singlet-triplet energy gap (Δ*E*_ST_). Therefore, the up-converted reverse intersystem crossing (RISC) from T_1_ to S_1_ is an endothermic process, which may lead to the quenching of long-lived triplet excitons in electroluminescence, and subsequently the reduction of device efficiency. Interestingly, organic molecules with inverted singlet-triplet (INVEST) gaps in violation of Hund’s multiplicity rule have recently come into the limelight. The unique feature has attracted extensive attention in the fields of organic optoelectronics and photocatalysis over the past few years. For an INVEST molecule possessing a higher T_1_ with respect to S_1_, namely a negative Δ*E*_ST_, the down-converted RISC from T_1_ to S_1_ does not require thermal activation, which is possibly conducive to solving the problems of fast efficiency roll-off and short lifetime of organic light-emitting devices. By virtue of this property, INVEST molecules are recently regarded as a new generation of organic light-emitting materials. In this review, we briefly summarized the significant progress of INVEST molecules in both theoretical calculations and experimental studies, and put forward suggestions and expectations for future research.

## Introduction

**FIGURE 1:**
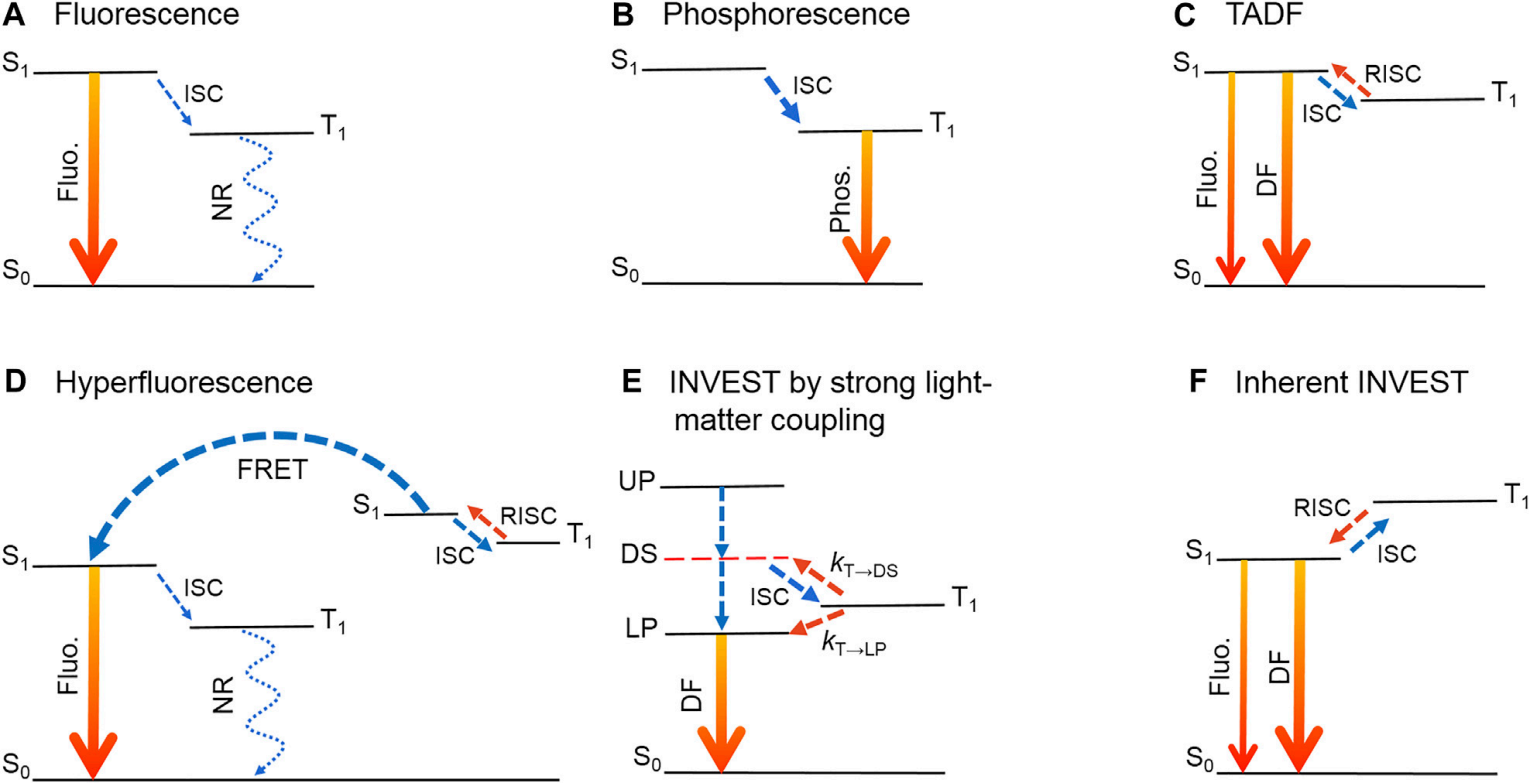
Luminescence mechanisms of organic light-emitting materials. **(A)** Fluorescence. **(B)** Phosphorescence. **(C)** Thermally activated delayed fluorescence (TADF). **(D)** Hyperfluorescence. **(E)** INVEST by strong light-matter coupling. **(F)** Inherent INVEST. Fluo., fluorescence; Phos., phosphorescence; DF, delayed fluorescence; ISC, intersystem crossing; RISC, reverse intersystem crossing; FRET, Förster resonance energy transfer; UP, upper polaritons; DS, dark singlet states; LP, lower polaritons; NR, nonradiative decay. *k*_T→DS_ and *k*_T→LP_: RISC rate constants from T_1_ to DS and from T_1_ to LP, respectively.

Organic light-emitting diodes (OLEDs) based on organic molecules have shown great prospects in the fields of solid-illuminations and displays by virtue of a number of advantages, such as autoluminescence, flexibility, high color purity and low power consumption (Hong et al., 2021). Over the past few decades, several luminescence mechanisms have been proposed, including fluorescence (Friend et al., 1999; Huang et al., 2012), phosphorescence (Bernhard et al., 2002; Minaev et al., 2014; Zhou et al., 2014), thermally activated delayed fluorescence (TADF) (Endo et al., 2011; Uoyama et al., 2012; Zhang et al., 2012; Li et al., 2013; Peng et al., 2020; Hung et al., 2022; Lv et al., 2022) and hyperfluorescence (Nakanotani et al., 2014; Chan et al., 2021). Fluorescent materials are commonly derived from pure organic molecules with stable luminescence properties and rapid radiative decay from the lowest excited singlet states (S_1_) to the singlet ground state (S_0_) (Figure 1A). According to spin statistics, the ratio of singlet and triplet excitons is about 1:3 under electrical excitation (Rothberg and Lovinger, 1996). Therefore, the maximum internal quantum efficiency (IQE) of a fluorescent OLED is only 25%, and consequently the external quantum efficiency (EQE) is limited to about 5%. In turn, the IQEs of phosphorescent OLEDs can theoretically reach 100% by capturing both singlet and triplet excitons as a consequence of strong spin-orbit coupling (SOC) effect induced by heavy atoms (Figure 1B) (Bernhard et al., 2002; Chi and Chou, 2010; Zhou et al., 2014; Mao et al., 2021). Nonetheless, the utilization of precious metals brings problems of high cost and environmental pollution. In this regard, researchers have to turn attention back to pure organic molecules, and extensive efforts harvesting triplets have been carried out. Among them, TADF has received tremendous attention since Endo et al. applied a pure organic molecule with the TADF character into an OLED (Endo et al., 2011). For a TADF molecule, a small energy difference (Δ*E*_ST_) between S_1_ and the lowest triplet excited state (T_1_) is required, which converts triplet excitons into singlet excitons through reverse intersystem crossing (RISC) (Figure 1C). Therefore, the IQEs of TADF emitters can also reach 100%. Meanwhile, Δ*E*_ST_ is proportional to the exchange integral between the spatial wave functions of the highest occupied molecular orbital (HOMO) and the lowest unoccupied molecular orbital (LUMO) (Endo et al., 2011; Yang et al., 2017). In this regard, separated Frontier orbital distributions are of significant importance during the molecular design of TADF materials (Fan et al., 2016).

Over the past decade, TADF molecules have been widely studied in light of the merits of high efficiency as well as low cost, and a number of TADF emitters have been developed (Jeon et al., 2019; Li et al., 2021c). Up to now, there are several pathways to realize TADF, such as traditional single molecule-based TADF (Uoyama et al., 2012; Zhang et al., 2012; Li et al., 2021d), exciplex-based TADF (Goushi et al., 2012; Li et al., 2014a; Oh et al., 2015; Li et al., 2021a; Li et al., 2021b; Xue and Xie, 2021; Li et al., 2022c; Gu et al., 2022), aggregation-induced emission (AIE)-based TADF (Zhao et al., 2018; Liu et al., 2020), excited-state intramolecular proton transfer (ESIPT)-based TADF (Mamada et al., 2017; Long et al., 2020) and multiple resonance-based TADF (MR-TADF) (Lee et al., 2020; Stavrou et al., 2021; Wu et al., 2021; Yang et al., 2022; Zou et al., 2022). Particularly, MR-TADF molecules have been considered as the most promising TADF materials on account of the attainment of both high efficiencies and high color purity. Nevertheless, the molecular design of MR-TADF is still rather limited in view of that almost all the MR-TADF molecules are B-, N-, S-, O-, carbonyl-, and/or sulfuryl-containing heterocyclic derivatives (Hatakeyama et al., 2016; Liang et al., 2018; Li et al., 2019; Yuan et al., 2019; Hall et al., 2020; Xu et al., 2021a; Huang et al., 2021; Meng et al., 2022). Additionally, for all the TADF molecules, there is always a problem that the RISC process is fairly slow, resulting in serious annihilation of triplet excitons and concomitantly serious efficiency roll-off at high current densities (Yersin et al., 2019; Hong et al., 2022).

Hyperfluorescence combines advantages of both fluorescence and TADF, in which fluorescent and TADF materials are introduced as emitters and host materials, respectively (Nakanotani et al., 2014). Under electrical excitation, almost all the singlet and triplet excitons are initially harvested by TADF molecules, and then triplet excitons can efficiently up-convert to be singlet excitons (Figure 1D). Subsequently, the energy can be transferred from S_1_ of TADF molecules to S_1_ of fluorescent molecules through Förster resonance energy transfer (FRET), and finally highly efficient fluorescence could be achieved (Chan et al., 2021). Notably, efficiency roll-off at high current densities could possibly occur in terms of a slow RISC in TADF molecules (Jakoby et al., 2019). Moreover, some other mechanisms, such as triplet-triplet annihilation (TTA) (Fukagawa et al., 2012; Jankus et al., 2013), pure organic room-temperature phosphorescence (RTP) (Zhou et al., 2018; Wen et al., 2021; Liu et al., 2022), utilization of higher excited states (Sato et al., 2015; Xu et al., 2021b), direct singlet harvesting (Yersin et al., 2019), doublet energy transfer with organic radicals (Li et al., 2022a) and radical-based emitters (Ai et al., 2018; Abdurahman et al., 2020; Cui et al., 2020), are proposed in recent years. However, current research suggests that these mechanisms have not yet shown a subversive improvement effect.

Recently, a mechanism of inverted singlet-triplet (INVEST) in violation of Hund’s multiplicity rule attracted much attention (Kollmar and Staemmler, 1978; Koseki et al., 1985; Segal et al., 2007; Difley et al., 2008; Sato et al., 2015; Ehrmaier et al., 2019; Gan et al., 2019; Mei et al., 2021; Pollice et al., 2021; Li et al., 2022b). For an INVEST molecule possessing a negative Δ*E*_ST_, the intrinsic photophysics of RISC are thereby completely overturned from an endothermic process to an exothermic one. Therefore, the RISC process of INVEST molecules are mostly likely superior to the corresponding TADF molecules. Consequently, INVEST emitters can theoretically outperform all previous generations in terms of considerably lower triplet exciton populations, and potential applications in OLEDs, organic lasers and photocatalysis could be imagined (Hwang and Schlenker, 2021; Pollice et al., 2021). At present, there are mainly two INVEST mechanisms, INVEST by strong light-matter coupling and inherent INVEST.

## Inverted singlet-triplet by strong light-matter coupling

INVEST by strong light-matter coupling is to build an optical microcavity to convert singlet excitons into two types of polaritons, namely lower polaritons (LPs) and upper polaritons (UPs). Meanwhile, an INVEST structure with a lower LP state relative to T_1_ can be realized by adjusting the microcavity structure (Figure 1E) (Eizner et al., 2019). Herein, polaritons are light-matter eigenstates forming when singlet electronic transitions are strongly coupled with the vacuum electromagnetic field in an optical cavity (Hopfield, 1958). In recent years, organic polaritons have been widely investigated in the areas of nonlinear interactions (Daskalakis et al., 2014), optoelectronic devices (Tischler et al., 2005; Ballarini et al., 2013; Sanvitto and Kena-Cohen, 2016; Eizner et al., 2018; Stranius et al., 2018) as well as chemical reactions (Feist et al., 2018; Ribeiro et al., 2018). Meanwhile, strong light-matter coupling has been regarded as an important tool to tailor molecular photophysical and photochemical properties without modifying chemical structures (Lidzey et al., 1998; Hertzog et al., 2019). As shown in Figure 1E, under optical excitation, the generation of delayed fluorescence for a TADF molecule in a polariton setup involves intersystem crossing (ISC) from the dark singlet state (DS) to T_1_, followed by two RISC processes from T_1_ to DS and from T_1_ to LP with rate constants of *k*_T→DS_ and *k*_T→LP_, respectively. In this situation, it is anticipated that the down-converted RISC process from T_1_ to LP could be dominant and conductive to the reduction of efficient roll-off if the *k*_T→LP_ can be much larger than *k*_T→DS_ by modifying the microcavity structure.

**FIGURE 2:**
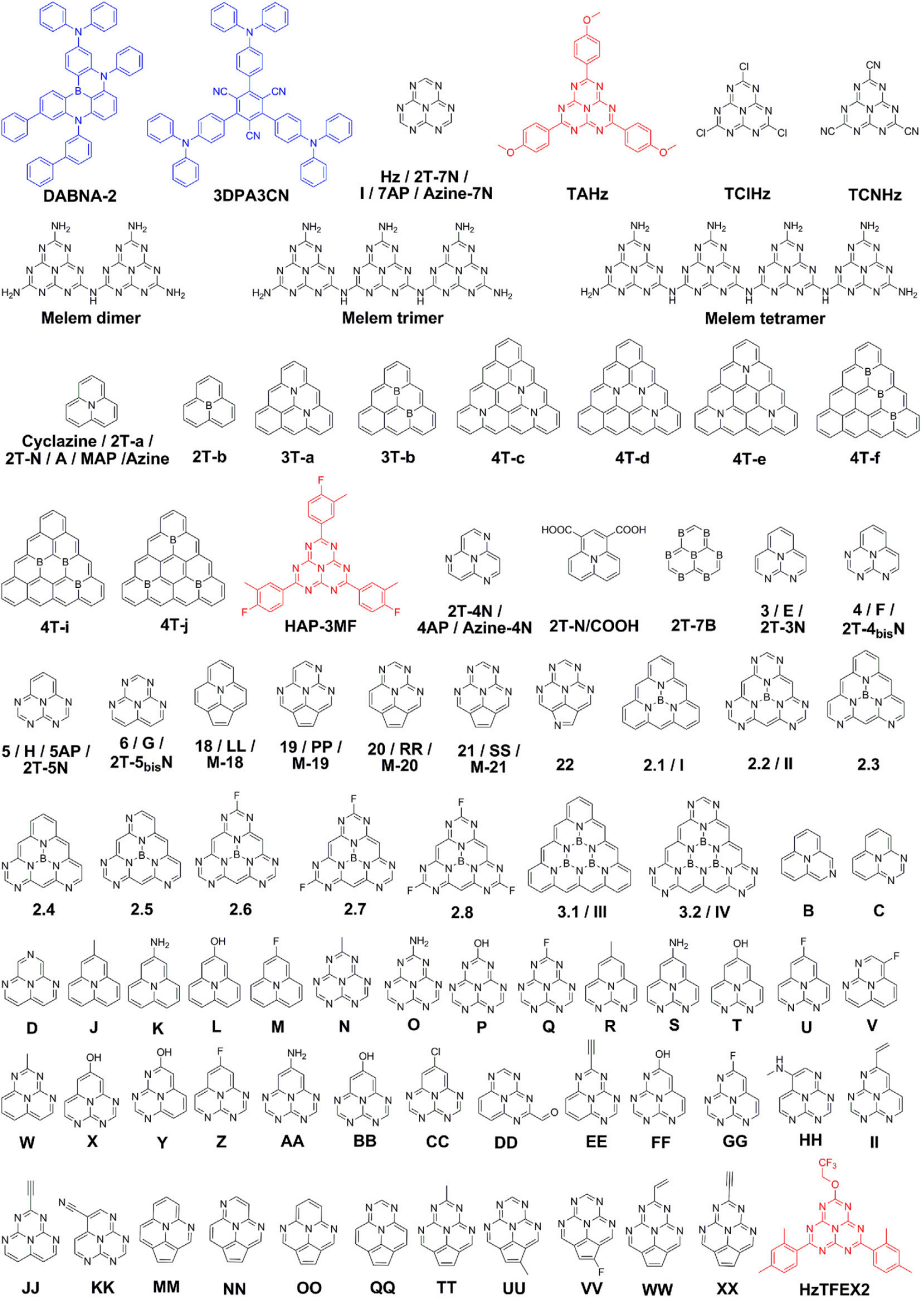
Chemical structures of INVEST molecules. The blue color represents INVEST molecules by strong light-matter coupling, while the black and red colors represent inherent INVEST molecules investigated by theoretical calculations and experimental verifications, respectively.

In 2019, Eizner et al. demonstrated an inversion of the singlet LP and T_1_ based on a TADF molecule, 1,3,5-tris(4-(diphenylamino)phenyl)-2,4,6-tricyanobenzene (3DPA3CN) (Figure 2), and measured the RISC rate constant in strongly coupled organic microcavities (Eizner et al., 2019). Unexpectedly, the RISC rate constants were almost invariable regardless of the large energy level shifts under strong light-matter coupling. In 2021, Yu et al. (2021) demonstrated a barrier-free RISC from a molecular centered triplet state to a hybrid polaritonic state based on another TADF molecule, 9-([1,1′-biphenyl]-3-yl)-*N*,*N*,5,11-tetraphenyl-5,9-dihydro-5,9-diaza-13b-boranaphtho[3,2,1-*de*]anthracen-3-amine (DABNA-2) (Figure 2), in light of a good compromise on Δ*E*_ST_ and coupling strength (Yu et al., 2021). Interestingly, the connection between the uncoupled T_1_ and the polaritonic state was shown to depend on molecular constitution of the polaritons. As the photonic nature of LP increased, a gradual disconnection from T_1_ happened. By choosing an intermediate state, a system with both an energetic driving force and enough molecular constitution of the LP was achieved to maintain a barrier-free RISC directly from T_1_ to LP. Accordingly, strong light-matter coupling offers a new strategy to overcome the limit of Hund’s rule and to facilitate the harvest of triplet excitons. From these results, it is anticipated that more efforts on INVEST by strong light-matter coupling will be extensively explored.

## Inherent inverted singlet-triplet

In addition to INVEST by strong light-matter coupling, inherent INVEST is another important strategy to realize singlet-triplet inversion and has drawn growing interest in the fields of organic optoelectronics and photochemistry (Ehrmaier et al., 2019; Miyajima et al., 2021; Pollice et al., 2021). Contrary to the vast majority of known organic molecules, inherent INVEST molecules possess an inherent property of singlet-triplet inversion without any assistance of the environment. Thus, energy transition from T_1_ to S_1_ is a spontaneous down-conversion process, replacing the up-conversion in TADF emitters (Figure 1F) (Pollice et al., 2021). Therefore, inherent INVEST are most likely to have better performance and applications with respect to INVEST by strong light-matter coupling.

Up to now, a series of inherent INVEST molecules have been theoretically or experimentally investigated, and the related chemical structures mentioned below are depicted in Figure 2. In 2019, Ehrmaier et al. (2019) investigated the excited singlet and triplet states of a set of heptazine derivatives (Hz, TAHz, TClHz, TCNHz, melem dimer, melem trimer and melem tetramer, Figure 2), by correlated ADC(2), CC2, EOM-CCSD, and CASPT2 calculations. Remarkably, all the heptazine derivatives displayed negative Δ*E*_ST_ from −0.30 to −0.20 eV with the ADC(2) method, indicating that the singlet-triplet inversion characteristics of heptazine derivatives were extremely robust, being affected neither by substitutions nor by oligomerization. Almost at the same time, de Silva theoretically studied the excited-state energy inversion of S_1_ and T_1_ based on a N-containing heterocycle, cyclazine (Figure 2), from the perspective of electronic structure theory (de Silva, 2019). Through systematic calculations and analyses with different excited-state electronic structure methods, it was found that electron correlation in the form of double excitations would lead to the reduction of Δ*E*_ST_ and the emergence of negative Δ*E*_ST_. The result indicates that popular electronic structure methods without the consideration of doubly excited configurations cannot accurately describe excited states of inherent INVEST molecules.

Notably, both abovementioned heptazine derivatives and cyclazine possess relatively small oscillator strengths, which are not qualified as emitters for highly efficient OLEDs. In 2021, Sanz-Rodrigo et al. (2021) calculated a set of N- and/or B-substituted triangle-shaped molecules by using popular time-dependent density functional theory (TD-DFT) and more sophisticated *ab initio* methods with correlation effects. Excitingly, molecules 2T-a, 2T-b, 3T-a, 3T-b, 4T-c, 4T-d, 4T-e, 4T-f, 4T-i and 4T-j (Figure 2) possess inherent INVEST characteristics and most of them showing nonvanishing oscillator strengths with highly correlated SA-CASSCF, SC-NEVPT2 and SCS-CC2 calculations (Sanz-Rodrigo et al., 2021). Sobolewski and Domcke investigated the electronic excitation energies of two previousy reported heptazine derivatives (HAP-3MF and HAP-3TPA) (Li et al., 2013; Li et al., 2014b) with the ADC(2) method, and HAP-3MF with a negative Δ*E*_ST_ of −0.24 eV was robustly verified (Sobolewski and Domcke, 2021). Dinkelbach et al. (2021) carried out a comprehensive theoretical study on the photophysics of Hz and HAP-3MF. Remarkably, they found that the ultimate luminescence efficiencies of these two inherent INVEST compounds were determined by not merely ISC/RISC processes but also the internal conversion from S_1_ to S_0_.

Ricci et al. (2021) assessed the excited-state energy order of a set of N- or B-doped π-conjugated heterocycles by linear-response TD-DFT and correlated *ab initio* methods. Among these molecules, negative Δ*E*_ST_ could be realized for molecules 2T-N, 2T-4N, 2T-7N, 2T-N/COOH and 2T-7B (Figure 2) by CIS(D), SCS-CC2, SCS-ADC(2) or SC-NEVPT2 methods. Noteworthily, they found that negative Δ*E*_ST_ should be ascribed to an intricate interplay between the singlet-triplet exchange interaction, the influence of doubly-excited configurations, and the impact of dynamic correlation effects. The result is of significant importance for further molecular design of inherent INVEST molecules. Pollice et al. (2021) put forward that ideal emitters potentially surpassing TADF materials should have both negative Δ*E*_ST_ and substantial fluorescence rates. Based on computational studies on a series of N-substituted phenalene derivatives, molecules possessing both negative singlet-triplet gaps and considerable fluorescence rates, namely 3–6 and 18–22 (Figure 2), were obtained, suggesting that inherent INVEST molecules are more common than hypothesized previously and have the potential to become the next generation organic light-emitting materials. Pios et al. (2021) designed and characterized a number of triangular boron carbon nitrides (2.1–2.8 and 3.1–3.2, Figure 2) conceptually derived from cyclazine and heptazine by employing high-level *ab initio* electronic structure theory. As expected, these compounds showed robust inherent INVEST characteristics, exhibiting great potential as chromophores for organic optoelectronics.

In 2022, Li and coworkers theoretically investigated the response of INVEST behavior of cyclazine to a static electric-field as well as an unchirped and chirped laser pulse by using next-generation quantum theory of atoms in molecules (NG-QTAIM), demonstrating that NG-QTAIM is a useful tool for understanding the response to laser irradiation (Li et al., 2022d). Recently, Alipour and Izadkhast comprehensively calculated a series of inherent INVEST emitters (A-Z, AA-XX and I-IV, Figure 2) toward the development of modern double-hybrid density functionals for singlet-triplet inversion (Alipour and Izadkhast, 2022). They found that particular proportions among the nonlocal exchange and correlation contributions as well as the same-spin and opposite-spin parameters included in the direct and indirect terms are needed to achieve a reliable accuracy for the singlet-triplet inversion. Sancho-Garcia et al. assessed the singlet-triplet inversion feature of a set of azaphenalene compounds (2T-N, 2T-4N, 2T-7N, 2T-3N, 2T-4_bis_N, 2T-5N and 2T-5_bis_N, Figure 2) by TD-DFT employing a family of double-hybrid density functionals, and found that double-hybrid exchange-correlation functionals incorporating double excitations could be a good alternative to wavefunction methods (Sancho-Garcia et al., 2022). Subsequently, Sancho-García and San-Fabián investigated four azaphenalene derivatives (MAP, 4AP, 5AP and 7AP) to assess if methods going beyond standard TD-DFT could predict accurate excited-state energy inversion (Sancho-García and San-Fabián, 2022). Interestingly, negative Δ*E*_ST_ with high accuracy could be well realized by employing methods merging wavefunction and correlation functionals. Moreover, Ghosh and Bhattacharyya calculated seven azaphenalene derivatives (M-18 to M-21, Azine, Azine-4N and Azine-7N, Figure 2) by combining DFT and wave function methods, unveiling that inherent INVEST gaps could be obtained by using doubles-corrected TD-DFT with suitable double-hybrid functionals or excited-state DFT (Ghosh and Bhattacharyya, 2022). Overall, the molecular design of inherent INVEST emitters should consider both minimal exchange integrals leading to small singlet-triplet gaps, and significant double excitation character in electronic transitions in extended π-conjugated heteroatom-containing molecular systems. Particularly, current inherent INVEST molecules are derived from N- and/or B-containing fused heterocycles.

Notably, present research on inherent INVEST is mainly carried out by theoretical calculations, while experimental explorations are fairly rare, possibly due to the difficulty in synthesis of these N- and/or B-containing fused heterocycles. Excitingly, Miyajima and coworkers experimentally demonstrated the existence of highly efficient inherent INVEST emitters for OLEDs (Miyajima et al., 2021). Based on computational screening on a large quantity of heptazine derivatives initially by affordable standard linear-response TD-DFT calculations and then by high-cost correlated wave function theories including double excitation configurations, a heptazine derivative, HzTFEX_2_ (Figure 2) was chosen for experimental evaluation considering both the possibility to be an efficient blue inherent INVEST emitter and the synthetic feasibility. Expectedly, HzTFEX_2_ showed a negative Δ*E*_ST_ of −11 meV based on the fit of Arrhenius equation, and meanwhile the rate inversion of RISC and ISC. Ultimately, an OLED incorporating HzTFEX_2_ exhibited a fairly high EQE of 17.0% with a fast transient electroluminescence decay. Recently, Li et al. (2022b) experimentally investigated the photophysical properties of HAP-3MF which was previously theoretically evaluated as the first inherent INVEST emitter (Sobolewski and Domcke, 2021). Surprisingly, negative Δ*E*_ST_ of −0.22 eV in toluene and −0.19 eV in acetonitrile were directly obtained from the fluorescence and phosphorescence spectra. Moreover, to reveal the extremely weak delayed emission, a mixed solution system of HAP-3MF:1,3-di(9H-carbazol-9-yl)benzene (mCP) in toluene at various molar ratios was subtly designed. As expected, enhanced delayed emissions were achieved, and the efficient triplet-exciton harvesting process *via* a down-converted triplet-to-singlet channel was elucidated.

## Conclusion and outlook

In summary, we have provided an overview of organic molecules with singlet-triplet inversion characteristics stemming from strong light-matter coupling and inherent INVEST. As compared to fluorescence, phosphorescence, TADF and hyperfluorescence, INVEST molecules possessing intriguing excited-state features have attracted great attention especially in the field of organic electroluminescence. Although numerous research results show that inherent INVEST molecules have great potential to become a new generation of high-performance organic light-emitting materials, there are still two main problems: 1) Most studies on INVEST molecules are merely based on theoretical calculations, whilst experimental results are currently sparse. 2) Present molecular design of inherent INVEST molecules is relatively limited, in view of that almost all these molecules are N- and/or B-containing heterocycles, especially heptazine derivatives. In this respect, it could be envisioned that more endeavors on experimental verifications and diverse molecular design will be carried out, and we believe that organic INVEST molecules will show bright prospects in organic optoelectronics and photochemistry in future.
